# Supplementary material for: Distinct molecular characteristics and virulence profiles of carbapenem-resistant Acinetobacter baumannii, Escherichia coli, and Enterobacter cloacae isolated from patients with inborn errors of immunity
Source: Microbiol Spectr. 2025 Jun 12;13(8):e00281-25. doi: 10.1128/spectrum.00281-25 (PMC12323661; doi:10.1128/spectrum.00281-25)
Supplement: Supplemental material — Figure S1; Tables S1 to S3. [file spectrum.00281-25-s0001.docx]

**Supplemental File**

**Figure S1:** Heatmap of antibiotic resistance genes and virulence genes of all strains. A. Heatmap for CR-AB strains; B. Heatmap for CR-ECO strains; C. Heatmap for CR-ECL strains.


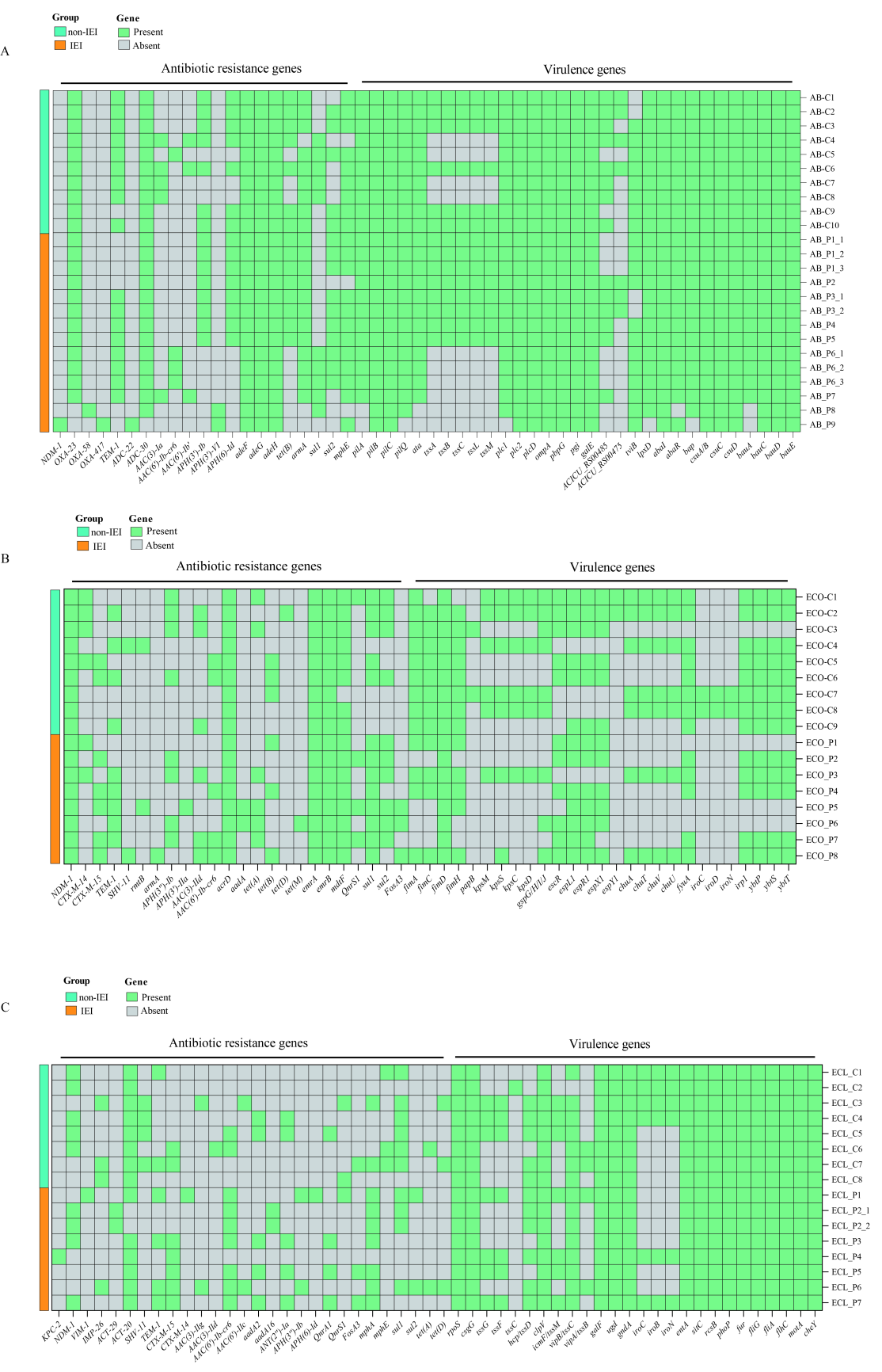


| Table S1 Genome assembly quality assessment of each strain. | | | | | | | |
| --- | --- | --- | --- | --- | --- | --- | --- |
| Sample ID | Clean reads | Mapped  (%) | Complete BUSCOs (%) | Contigs | GC(%) | N50 | Total length |
| AB_P1_1 | 12597150 | 99.21 | 98.40 | 68 | 38.80 | 145438 | 3959193 |
| AB_P1_2 | 7107586 | 99.53 | 98.40 | 69 | 38.81 | 135160 | 3957159 |
| AB_P1_3 | 12883532 | 99.51 | 98.40 | 64 | 38.81 | 162048 | 3959348 |
| AB_P2 | 3520258 | 99.63 | 98.40 | 65 | 38.84 | 100179 | 3947915 |
| AB_P3_1 | 6450422 | 99.50 | 98.40 | 72 | 38.84 | 159566 | 3976149 |
| AB_P3_2 | 18978280 | 99.61 | 98.40 | 72 | 38.84 | 160405 | 3976281 |
| AB_P4 | 6337568 | 99.79 | 98.40 | 63 | 38.82 | 157831 | 3928267 |
| AB_P5 | 23197230 | 99.58 | 98.40 | 63 | 38.82 | 156113 | 3885285 |
| AB_P6_1 | 20612514 | 99.58 | 98.40 | 67 | 38.85 | 146732 | 3919848 |
| AB_P6_2 | 13624974 | 99.64 | 98.40 | 65 | 38.85 | 146731 | 3919966 |
| AB_P6_3 | 14003420 | 99.61 | 98.40 | 66 | 38.85 | 146731 | 3920452 |
| AB_P7 | 13612172 | 99.74 | 98.40 | 68 | 38.83 | 178517 | 3962441 |
| AB_P8 | 14345816 | 99.62 | 97.60 | 156 | 38.87 | 62598 | 4138309 |
| AB_P9 | 12028676 | 99.68 | 99.20 | 136 | 38.96 | 76488 | 4012448 |
| AB_C1 | 17839506 | 99.70 | 98.40 | 60 | 38.84 | 162296 | 3927389 |
| AB_C2 | 19421396 | 99.63 | 98.40 | 77 | 38.86 | 162048 | 4022438 |
| AB_C3 | 15462516 | 99.70 | 98.40 | 64 | 38.82 | 160139 | 3924741 |
| AB_C4 | 21166164 | 99.75 | 98.40 | 69 | 38.86 | 114831 | 3811141 |
| AB_C5 | 15404440 | 99.78 | 98.40 | 69 | 38.85 | 146732 | 3920537 |
| AB_C6 | 3110204 | 99.90 | 98.40 | 86 | 38.86 | 112435 | 4023111 |
| AB_C7 | 30594032 | 99.75 | 98.40 | 69 | 38.84 | 166701 | 3974019 |
| AB_C8 | 9303970 | 99.90 | 98.40 | 78 | 38.81 | 157535 | 4009452 |
| AB_C9 | 13430402 | 99.63 | 98.40 | 64 | 38.80 | 144857 | 3932994 |
| AB_C10 | 24009876 | 99.71 | 98.40 | 61 | 38.82 | 157831 | 3926453 |
| ECO_P1 | 15929142 | 99.44 | 100.00 | 93 | 50.50 | 131700 | 4943017 |
| ECO_P2 | 7606590 | 99.58 | 99.20 | 133 | 50.64 | 93380 | 4730936 |
| ECO_P3 | 11735148 | 99.45 | 100.00 | 95 | 50.52 | 180519 | 5144474 |
| ECO_P4 | 6568912 | 99.57 | 100.00 | 113 | 50.49 | 113153 | 4913866 |
| ECO_P5 | 9863304 | 99.09 | 100.00 | 200 | 50.66 | 85621 | 5001554 |
| ECO_P6 | 13661792 | 99.57 | 100.00 | 158 | 50.37 | 119111 | 5206802 |
| ECO_P7 | 24552634 | 99.67 | 100.00 | 158 | 50.66 | 108434 | 4968355 |
| ECO_P8 | 2992446 | 99.61 | 100.00 | 151 | 50.45 | 101662 | 5406284 |
| ECO_C1 | 19142064 | 99.50 | 100.00 | 173 | 50.50 | 129094 | 5343797 |
| ECO_C2 | 10499616 | 99.58 | 100.00 | 111 | 50.59 | 150293 | 5201104 |
| ECO_C3 | 13598222 | 99.76 | 100.00 | 117 | 50.75 | 101826 | 4967386 |
| ECO_C4 | 16032302 | 99.78 | 100.00 | 118 | 50.73 | 161037 | 5183858 |
| ECO_C5 | 16065656 | 99.81 | 100.00 | 117 | 50.55 | 132886 | 4971458 |
| ECO_C6 | 14679466 | 99.78 | 100.00 | 100 | 50.53 | 160962 | 4881103 |
| ECO_C7 | 12961986 | 99.40 | 100.00 | 147 | 50.33 | 149069 | 5319538 |
| ECO_C8 | 23050976 | 99.86 | 100.00 | 45 | 50.45 | 218195 | 4832845 |
| ECO_C9 | 11298752 | 99.27 | 100.00 | 168 | 50.65 | 111577 | 5224949 |
| ECL_P1 | 8311882 | 99.70 | 98.40 | 84 | 55.17 | 149903 | 5046926 |
| ECL_P2_1 | 11670892 | 99.85 | 99.20 | 49 | 55.49 | 267366 | 4872376 |
| ECL_P2_2 | 8158146 | 99.88 | 99.20 | 44 | 55.49 | 280250 | 4871924 |
| ECL_P3 | 4360812 | 99.54 | 99.20 | 107 | 54.70 | 135856 | 4989679 |
| ECL_P4 | 2704178 | 99.72 | 98.40 | 63 | 55.38 | 208639 | 5018641 |
| ECL_P5 | 12091758 | 99.83 | 99.20 | 84 | 54.53 | 163498 | 5130335 |
| ECL_P6 | 2695344 | 99.55 | 98.40 | 130 | 54.82 | 159898 | 5030445 |
| ECL_P7 | 9206144 | 99.76 | 98.40 | 104 | 54.83 | 180663 | 5388339 |
| ECL_C1 | 23242774 | 99.36 | 98.40 | 70 | 55.19 | 237514 | 5039469 |
| ECL_C2 | 13191880 | 99.87 | 98.40 | 34 | 55.53 | 219242 | 4641044 |
| ECL_C3 | 9964104 | 99.76 | 98.40 | 64 | 54.84 | 208645 | 5228801 |
| ECL_C4 | 25336248 | 99.78 | 98.40 | 74 | 54.88 | 160845 | 5230594 |
| ECL_C5 | 9570218 | 99.86 | 99.20 | 90 | 54.91 | 134705 | 4918177 |
| ECL_C6 | 9863556 | 99.59 | 98.40 | 95 | 55.14 | 132827 | 4776298 |
| ECL_C7 | 18475816 | 99.83 | 98.40 | 70 | 54.79 | 193617 | 4914591 |
| ECL_C8 | 10693156 | 99.38 | 98.40 | 77 | 55.31 | 167979 | 4758101 |
| Clean reads are high-quality sequencing reads obtained after removing low-quality data, adapters, and contaminants. Mapped (%) indicates the percentage of reads aligned to the reference genome. Complete BUSCOs (%) represent the percentage of complete Benchmarking Universal Single-Copy Orthologs (BUSCOs) in the assembly, reflecting genome completeness. Contigs are continuous DNA sequences assembled from raw reads. GC (%) denotes the percentage of guanine (G) and cytosine (C) bases in the DNA sequence. N50 represents the length of the shortest contig at which the cumulative length of all longer or equal-length contigs reaches 50% of the total assembly size，serving as a measure of assembly quality.Total length refers to the overall length of the assembled contigs. | | | | | | | |

| Table S2 Detailed immunophenotype of patients with IEI. | | | | | | | | | | | | |
| --- | --- | --- | --- | --- | --- | --- | --- | --- | --- | --- | --- | --- |
| Patient ID | Gender | Age (M) | T (cells/uL) | B (cells/uL) | NK (cells/uL) | IgA (g/L) | IgG (g/L) | IgM (g/L) | IgE (kU/L) | DHR SI | Gene mutation | Clinical manifestation |
| AB_P1 | M | 8.77 | 1883.20 | 1736.24 | 579.30 | 0.47 | 8.90 | 0.44 | <2.0 | Decreased | *CYBB* | Recurrent infections |
| AB_P2 | M | 2.43 | 1498.40 | 55.39 | 170.71 | 0.13 | 6.70 | 0.36 | 8.57 | Decreased | *CYBB* | Recurrent infections |
| AB_P3 | M | 120.00 | 532.70 | 40.43 | 148.66 | 0.19 | 14.70 | 6.92 | 166.15 |  | *RAG1* | Recurrent pneumonia and skin infections |
| AB_P4 | M | 9.20 | 1498.00 | 55.39 | 170.71 | 0.21 | 6.40 | 0.53 | 25.47 | Decreased | *CYBB* | Recurrent respiratory tract infections |
| AB_P5 | M | 4.13 | 2.20 | 533.24 | 105.23 | 0.01 | 6.90 | 0.10 | 4.17 |  | *IL2RG* | Recurrent infections |
| AB_P6 | M | 4.13 | 732.00 | 107.89 | 263.70 | 0.27 | 8.20 | 4.95 | 108.4 |  | Unknown | Recurrent pneumonia |
| AB_P7 | F | 17.00 | 274.50 | 1363.27 | 157.74 | 0.34 | 23.60 | 0.68 | 40.45 |  | *RAG1* | Skin infections |
| AB_P8 | M | 204.00 | 600.30 | 363.74 | 472.57 | 1.32 | 16.20 | 0.59 | 8374.86 |  | *DOCK8* | Recurrent skin rashes |
| AB_P9 | M | 24.00 | 498.00 | 313.00 | 402.00 | 0.23 | 15.80 | 0.45 | 0.72 |  | *RAG1* | Recurrent skin rashes and infections |
| ECO_P1 | M | 2.40 | 2949.10 | 520.87 | 592.80 | 0.12 | 8.00 | 1.03 | 110.4 | Decreased | *CYBB* | Severe pneumonia |
| ECO_P2 | M | 10.00 | 1344.30 | 314.47 | 432.71 | 0.08 | 6.70 | 0.46 | 14.39 | Decreased | *CYBB* | Recurrent infections |
| ECO_P3 | M | 7.33 | 1012.10 | 1120.91 | 269.81 | 0.29 | 10.50 | 2.69 | 18.01 | Decreased | *CYBB* | Recurrent skin infections |
| ECO_P4 | M | 9.00 | 783.00 | 1031.58 | 97.16 | 0.68 | 13.40 | 1.32 | 58.24 | Decreased | *CYBB* | Recurrent infections |
| ECO_P5 | M | 40.00 | 3488.90 | 861.97 | 186.57 | 0.09 | 10.40 | 0.14 | 98.46 |  | *NFKBIA* | Recurrent skin rashes and infections |
| ECO_P6 | F | 28.00 | 1314.10 | 33.14 | 249.98 | 1.11 | 10.30 | 0.36 | 38.66 |  | *RAG1* | Recurrent infections |
| ECO_P7 | M | 144.00 | 719.40 | 388.33 | 189.88 | 1.47 | 14.20 | 2.23 | 94.13 | Decreased | *CYBB* | Recurrent **pneumonia** |
| ECO_P8 | M | 27.00 | 1900.00 | 1457.33 | 331.27 | 3.36 | 23.20 | 3.21 | 914.32 |  | *ELANE* | Recurrent respiratory tract infections |
| ECL_P1 | M | 8.57 | 2571.50 | 1675.02 | 347.28 | 0.27 | 8.40 | 1.82 | 24.03 | Decreased | *CYBB* | Recurrent skin infections |
| ECL_P2 | M | 8.90 | 2528.60 | 2699.97 | 94.43 | 0.66 | 14.10 | 1.95 | 968.16 | Decreased | *CYBB* | Recurrent infections |
| ECL_P3 | M | 25.00 | 1363.50 | 1035.09 | 667.22 | 0.33 | 6.00 | 1.33 | 46.17 | Decreased | *CYBB* | Recurrent infections |
| ECL_P4 | M | 48.00 | 64.20 | 11.64 | 42.99 | 0.01 | 6.40 | 0.57 | 14.16 |  | Unknown | Recurrent infections |
| ECL_P5 | M | 10.90 | 1435.00 | 1316.06 | 408.74 | 0.72 | 10.10 | 1.71 | 52.98 | Decreased | *CYBB* | Recurrent **pneumonia** |
| ECL_P6 | M | 19.00 | 2598.80 | 3.33 | 116.43 | 0.17 | 13.50 | 0.34 | <2.0 |  | Unknown | Recurrent infections |
| ECL_P7 | M | 48.25 | 888.70 | 290.30 | 154.04 | 0.31 | 11.70 | 1.87 | 39.74 | Decreased | Unknown | Recurrent infections |
| M, male; F, female；DHR, dihydrorhodamine; SI, stimulation index. | | | | | | | | | | | | |

| Table S3 Clinical characteristics of patients without IEI. | | | | |
| --- | --- | --- | --- | --- |
| Patient ID | Gender | Age (M) | Isolation sites | Diagnosis |
| AB_C1 | M | 84.00 | Bloodstream | Sepsis |
| AB_C2 | M | 18.00 | Respiratory tract | Hydrocephalus, Pneumonia |
| AB_C3 | M | 24.00 | Respiratory tract | Sepsis, Pneumonia |
| AB_C4 | F | 8.08 | Respiratory tract | Encephalitis, Pneumonia |
| AB_C5 | F | 4.67 | Respiratory tract | Hypoglycemia, Pneumonia |
| AB_C6 | M | 2.92 | SSTIs | Encephalitis, Skin infection |
| AB_C7 | M | 4.42 | Bile | Liver injury |
| AB_C8 | F | 1.92 | Bloodstream | Sepsis, Pneumonia |
| AB_C9 | M | 26.00 | Respiratory tract | Epilepsy, Pneumonia |
| AB_C10 | M | 38.00 | SSTIs | Gastrointestinal perforation, Skin infection |
| ECO_C1 | F | 1.50 | Urinary tract | Pneumonia, Urinary tract infection |
| ECO_C2 | F | 108.00 | Urinary tract | Septicemia, Urinary tract infection |
| ECO_C3 | M | 6.03 | Urinary tract | Vesicoureteral reflux |
| ECO_C4 | M | 16.00 | Urinary tract | Hydronephrosis |
| ECO_C4 | M | 16.00 | Urinary tract | Kawasaki disease, Urinary tract infection |
| ECO_C6 | M | 8.56 | Respiratory tract | Pneumonia |
| ECO_C7 | M | 5.23 | Urinary tract | Ureteral obstruction |
| ECO_C8 | M | 0.60 | Respiratory tract | Respiratory tract infection |
| ECO_C9 | M | 7.17 | Urinary tract | Hydronephrosis |
| ECL_C1 | F | 56.00 | Urinary tract | Gastrointestinal hemorrhage, Urinary tract infection |
| ECL_C2 | M | 12.37 | Urinary tract | Pneumonia, Urinary tract infection |
| ECL_C3 | F | 64.00 | SSTIs | Fracture, Sepsis |
| ECL_C4 | M | 108.00 | Urinary tract | Hydronephrosis |
| ECL_C5 | M | 11.00 | Urinary tract | Bronchitis, Urinary tract infection |
| ECL_C6 | M | 6.23 | Respiratory tract | Encephalitis, Pneumonia |
| ECL_C7 | M | 0.77 | Urinary tract | Urinary tract infection |
| ECL_C8 | F | 0.66 | Urinary tract | Urinary tract infection, Pneumonia |
